# MASPs at the crossroad between the complement and the coagulation cascades - the case for COVID-19

**DOI:** 10.1590/1678-4685-GMB-2020-0199

**Published:** 2021-03-17

**Authors:** Valéria Bumiller-Bini, Camila de Freitas Oliveira-Toré, Tamyres Mingorance Carvalho, Gabriela Canalli Kretzschmar, Letícia Boslooper Gonçalves, Nina de Moura Alencar, Miguel Angelo Gasparetto, Marcia Holsbach Beltrame, Angelica Beate Winter Boldt

**Affiliations:** 1 Universidade Federal do Paraná (UFPR), Departamento de Genética, Laboratório de Genética Molecular Humana, Curitiba, PR, Brazil.; 2 Universidade Federal do Paraná (UFPR), Departamento de Genética, Programa de Pós-Graduação em Genética, Curitiba, PR, Brazil.; 3 Universidade Federal do Paraná (UFPR), Programa de Pós-Graduação em Medicina Interna e Ciências da Saúde, Laboratório de Imunopatologia Molecular, Curitiba, PR, Brazil.; 4 Universidade Federal do Paraná, Departamento de Genética, Laboratório de Citogenética Humana e Oncogenética, Curitiba, PR, Brazil.; 5 Universidade Federal do Paraná (UFPR), Departamento de Genética, Laboratório de Imunogenética e Histocompatibilidade (LIGH), Curitiba, PR, Brazil.; 6 Fundação Oswaldo Cruz (Fiocruz), Instituto Carlos Chagas, Programa de Pós-Graduação em Biociências e Biotecnologia, Laboratório de Virologia Molecular, Curitiba, PR, Brazil.

**Keywords:** COVID-19, complement system, lectin pathway, MASP, coagulation

## Abstract

Components of the complement system and atypical parameters of coagulation were reported in COVID-19 patients, as well as the exacerbation of the inflammation and coagulation activity. Mannose binding lectin (MBL)- associated serine proteases (MASPs) play an important role in viral recognition and subsequent activation of the lectin pathway of the complement system and blood coagulation, connecting both processes. Genetic variants of *MASP1* and *MASP2* genes are further associated with different levels and functional efficiency of their encoded proteins, modulating susceptibility and severity to diseases. Our review highlights the possible role of MASPs in SARS-COV-2 binding and activation of the lectin pathway and blood coagulation cascades, as well as their associations with comorbidities of COVID-19. MASP-1 and/or MASP-2 present an increased expression in patients with COVID-19 risk factors: diabetes, arterial hypertension and cardiovascular disease, chronic kidney disease, chronic obstructive pulmonary disease, and cerebrovascular disease. Based also on the positive results of COVID-19 patients with anti-MASP-2 antibody, we propose the use of MASPs as a possible biomarker of the progression of COVID-19 and the investigation of new treatment strategies taking into consideration the dual role of MASPs, including MASP inhibitors as promising therapeutic targets against COVID-19.

## Introduction

The emergence of a new infectious agent late in 2019 brought back the attention of the world to the Coronaviridae family (reviewed by Hui *et al*., 2020; Malik, 2020). In 2003, SARS emerged as a highly infectious disease caused by SARS-CoV-1 in Asia (reviewed by Yang *et al*., 2020). The group of positive-sense RNA viruses took the central spotlight in science and society, after severe acute respiratory syndrome coronavirus 2 (SARS-CoV-2) was identified as the causative agent of the respiratory disease, known as Coronavirus Disease 2019 (COVID-19) (reviewed by Brian and Baric, 2005; Zhou *et al*., 2020c).

In March of 2020, the World Health Organization (WHO) proclaimed a pandemic caused by SARS-CoV-2 (reviewed by Valencia, 2020). COVID-19 ranges from asymptomatic to severe cases and may result in death. The estimated proportion of asymptomatic individuals ranges from 20.6 to 39.9%, with an all-age infectious fatality rate for COVID-19 of 1.3% (Mizumoto *et al*., 2020; Russell *et al*., 2020). In Brazil to date, there are 5,055,888 confirmed cases and 149,639 deaths reported (WHO, 2020a).

SARS-CoV-2 is a positive single-stranded nonsegmented RNA virus with its genome comprised under an envelope covered by glycoproteins, which are potential targets for lectin receptors of the innate immune response (reviewed by Jin *et al*., 2020). The virus is transmitted primarily via respiratory droplets. Once in contact with the epithelial cell of the airways, the interaction between the envelope spike viral glycoprotein (protein S) and components of host cell, such as angiotensin-converting enzyme 2 receptor (ACE-2) and transmembrane serine protease 2 (TMPRSS2), allows it to enter the cell and initiate the viral replicative cycle (Hoffmann *et al*., 2020; Zhou *et al*., 2020c). Although particular sites on its glycoprotein spikes are mutated compared to SARS-CoV-1, SARS-CoV-2 uses the same entry receptor and recognizes it with a similar affinity (Shang *et al*., 2020).

As observed in other coronaviruses infections, the host immune response plays an essential role in defeating the infection. However, it is also associated with tissue damage and disease severity (reviewed by Rabaan *et al*., 2020). The glycoproteins on the membrane of SARS-CoV-2 represent pathogen-associated molecular patterns (PAMPs), recognized by pattern-recognition receptors (PRRs) of the innate immune response. This response appears to be crucial in the early stages and progression of the disease. Initial innate responses are also based on humoral components, such as cytokines, innate antibodies, and proteins of the coagulation and complement cascades (Matricardi *et al*., 2020).

This response may eliminate the virus, or, out of control, cause tissue damage and reduced lung capacity (Li *et al*., 2020; Matricardi *et al*., 2020; Tay *et al*., 2020). In fact, damage of the respiratory tract in COVID-19 infection has been rather credited to a dysregulated immune response, than to an amplified viral replication. The activation of lung immune cells by PRRs promotes the release of pro-inflammatory cytokines, neutrophils, and monocytes into the bronchi. This may obstruct the air-blood barrier, causing tissue damage in airway epithelial and vascular endothelial cells, which also express ACE-2 (Risitano *et al*., 2020).

SARS-CoV-2 infection appears to have a delayed or suppressed Type I IFN response during initial infection, and viral replication triggers a hyperinflammatory state, culminating in a cytokine storm. Severe cases of COVID-19 require access to the intensive care unit. Infected individuals have shown increased levels of different cytokines related to innate immune response, especially interleukin-6 (IL-6) (reviewed by Liu *et al*., 2020; Prompetchara *et al*., 2020). IL-6 is mainly produced by activated monocytes and macrophages, as well as by the liver (reviewed by Tanaka *et al*., 2014). This pro-inflammatory cytokine activates pathways leading to an increased cytokine release, maturation of naïve T cell, alterations in vessel permeability, heart contractibility, and inflammatory proteins produced by the liver, including complement components (Li *et al*., 2020; Ye *et al*., 2020).

SARS-CoV-2 may activate the classical (CP) and lectin (LP) pathways of the complement system (CS) (Matricardi *et al*., 2020), and LP components were found deposited in lung tissue of COVID-19 patients (Gao *et al*., 2020b). The CS comprises more than 50 circulating proteins, cell surface receptors and regulators acting at the forefront of the host defense against microorganisms, including the coronaviruses (Ip *et al*., 2005; Gialeli *et al*., 2018; Gralinski *et al*., 2018). It detects and eliminates foreign bodies, clears immune complexes and cell debris, and connects the innate to the adaptive immune responses (reviewed by Ricklin *et al*., 2016; Gialeli *et al*., 2018). It is a proteolytic cascade, activated by three distinct pathways: CP, LP and alternative pathway (AP), which result in the recruitment of inflammatory cells, phagocytosis, neutrophil extracellular traps (NETs) and cell lysis through the membrane-attack complex (MAC, composed by C5b-C9 components) (Ricklin *et al*., 2016; Bont *et al*., 2019). Moreover, CS is well-connected with the immune surveillance system, and has an intense crosstalk with other responses, such as the coagulation cascade (reviewed by Ricklin *et al*., 2016). The dysregulation of complement activity, or its exacerbation, may contribute to tissue damage, leading to inflammatory and immunological diseases (reviewed by Ricklin *et al*., 2016; Jodele and Köhl, 2020).

All three complement pathways are initiated by different triggers. Depending on the concomitant presence of these triggers on the same surface, all may start simultaneously and compete for central (C3) or terminal (C5-C9) CS components. In this case, inhibition of one pathway may be compensated by another one, with more C3 and C5-C9 at disposal. PRRs with flexible recognition sites within the LP ensure its activation for most (but not all) triggers (reviewed by Beltrame *et al*., 2015; Ricklin *et al*., 2016; and Gialeli *et al*., 2018). This explains why people that present deficiency on one PRR of the LP may still present a working complement system, against most pathogens (Thiel *et al*., 2007; Ammitzbøll *et al*., 2013). LP and CP share the same physiological inhibitor (as C1-INH), whereas the alternative pathway depends on factors B and H for an efficient inhibition. Since AP mostly depends on spontaneous C3 hydrolysis, complement activation may still occur in the absence of the first two pathways. However, specific inhibition of the LP is not predicted to silence either pathway, neither to exacerbate them, excepting only the aforementioned case with multiple different immunological triggers. On the other hand, AP works as a positive feedback loop for either LP or CP, its inhibition is expected to slow down the CS amplification process (reviewed by Beltrame *et al*., 2015; Ricklin *et al*., 2016; and Gialeli *et al*., 2018).

**Figure 1 -:**
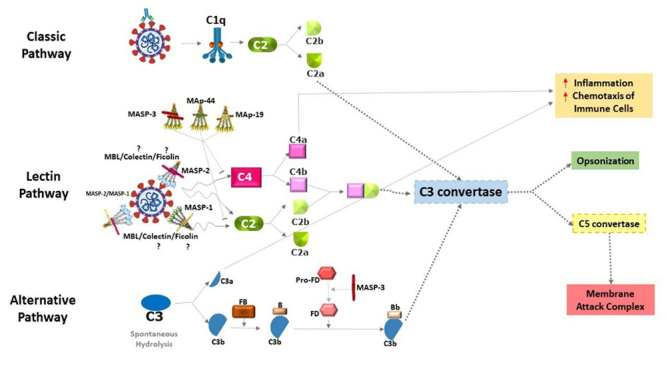
Complement pathways in SARS-CoV-2 infection. The activation of the classical pathway occurs through the C1 complex, after recognition of antibodies complexed to SARS-CoV-2. This leads to the cleavage of the C2 component into C2a and C2b. C2a joins the common pathway of the three complement pathways to form the C3 convertase. Activation of the lectin pathway by the virus via the MBL/MASP-1/MASP-2 complex has already been demonstrated, however the activation by ficolins or colectin is not shown (with a question mark). After binding of MBL/MASP complexes to the surface of pathogens, MASP-1 autoativates, transactivates MASP-2, and C2 and C4 components are cleaved (C2 and C4 by MASP-2 and C2 by MASP-1), generating the C3 convertase. The alternative pathway is initiated by the spontaneous hydrolysis of component C3, generating C3a and C3b. C3b binds to factor B and is cleaved by factor D, forming the C3 convertase of the alternative pathway. After this step, the three pathways converge into a single pathway. The C3 convertase enzyme cleaves component C3 into C3a and C3b. C3a and C4a are anaphylatoxins that contribute to an increase in inflammatory processes and to the chemotaxis of neutrophils and macrophages (red arrows), while C3b performs viral opsonization. The formation of C5 convertase occurs in different ways through the three pathways, but all generate C5a and C5b. C5a is an anaphylatoxin (as also C3a) that contributes to inflammatory processes, while C5b joins the last C6-C9 components of the cascade and forms the membrane attack complex. The MASP-3, MAp44, and probably MAp19 proteins inhibit the lectin pathway. MASP-3 also participates in the cleavage of pro-factor D into factor D, of the alternative pathway. The elements of the figure are not shown in their actual proportions.

In the case of COVID-19, the CP of complement can be activated, approximately, one week after virus incubation by the recognition of IgG/IgM-SARS-CoV-2 immune complexes (Wölfel *et al*., 2020). Yet, LP is the first pathway to get activated after the first contact with the virus, by the binding of mannose-binding lectin (MBL), associated with homodimers of MBL-associated serine proteases (MASPs), to viral N-Glycan or immunocomplexes formed by the IgA immunoglobulin and SARS-CoV-2 (Figure 1). Both activations can lead to cell damage (Matricardi *et al*., 2020; Jodele and Köhl, 2020).

C3a, C5a and MAC have been found at high levels in COVID-19 patients, increasing with severity of the disease, together with immunoglobulin IgG and C4 consumption (Gao *et al*., 2020b; Marcos-Jimenez *et al*., 2020; Skendros *et al*., 2020). C5a is a product of C5 cleavage and has a potent chemotactic response, inducing the migration of the neutrophils (Ehrengruber *et al*., 1994), and the formation of NETs (reviewed by Bont *et al*., 2019). NET release triggers AP activation through properdin binding (Yuen *et al*., 2016), and tissue-factor bearing NETs induced by COVID-19 platelet-rich plasma launched thrombotic activity of endothelial cells (Skendros *et al*., 2020), resulting in increased inflammation and coagulation activity. Furthermore, activation of the C5a receptor (C5aR1) leads to inflammasome activation (Jodele and Köhl, 2020). The MASPs of the LP may also activate platelets and tissue factors, favoring coagulation and microthrombosis. These events are particularly common in the clinical stage of acute respiratory failure, observed in severe cases of the disease (Matricardi *et al*., 2020).

Indeed, SARS-CoV-2 seems to affect the coagulation cascade (Chen *et al*., 2020b; Zhou *et al*., 2020b; Wang *et al*., 2020b; Tang *et al*., 2020a; reviewed by Connors and Levy, 2020; Zhang *et al*., 2020b). Atypical coagulation parameters associated with COVID-19 appeared in early reports from China (Wuhan). Of a total of 99 COVID-19 patients, 6% had increased activated partial thromboplastin time, 5% elevated prothrombin and 36% elevated D-dimer (a protein fragment resulting from clot dissolution) levels. Further, increased concentrations of IL-6 and C-reactive protein and a higher erythrocyte sedimentation rate were reported (Chen *et al*., 2020b; reviewed by Connors and Levy, 2020). High levels of D-dimer have been associated with increased mortality rates in COVID-19 (Zhou *et al*., 2020b; Tang *et al*., 2020b; Zhang *et al*., 2020b). Its measurement has been suggested as a routine test for an early marker of the disease (pre-print Zhang *et al*., 2020a).

In this review, we focus on the role of MASPs within the LP and coagulation pathways, and their possible association through genetic polymorphisms, protein levels, and isoforms with COVID-19 and comorbidities, increasing the risk for severe disease, highlighting MASPs as promising biomarkers of disease progression and also as therapeutic targets against COVID-19.

### MASPs and the lectin pathway

The lectin pathway can be activated by PRRs: collectins as MBL and collectin kidney 1 (CL-K1 or CL-11) or ficolins (ficolin-1 or M-ficolin, ficolin-2 or L-ficolin, and ficolin-3 or H-ficolin), associated to homodimers of MASPs (Fujita, 2002; Hansen *et al*., 2010; Kjaer *et al*., 2013). The PRRs recognize carbohydrate and acetylated residues on the surface of pathogens and altered cells. After binding, the MASP-1 zymogen autoactivates and transactivates MASP-2. MASP-2 further cleaves the C4 complement component, whereas both MASP-1 and MASP-2 cleave C2. This leads to the formation of the C3 convertase (C4bC2b), which is also produced by the initiation of the CP. The C3 convertase cleaves C3, a central component of the CS. This cleavage is amplified by the alternative pathway of complement and produces the C3b opsonin and the C3a anaphylatoxin. C3b binds to the target surface and results in its opsonization and phagocytosis, thereby promoting the presentation of antigens by phagocytes, initiating adaptive immune responses. The anaphylatoxins include C5a, produced in the final steps of the cascade. They are recognized by specific receptors and active inflammatory responses. The C5 convertase (C4bC2bC3b), subsequently cleaves C5, triggering formation of the membrane attack complex, resulting in cell lysis (Figure 1) (reviewed by Beltrame *et al*., 2015; Boldt *et al*., 2016b; Andrade *et al*., 2017).

**Figure 2 -:**
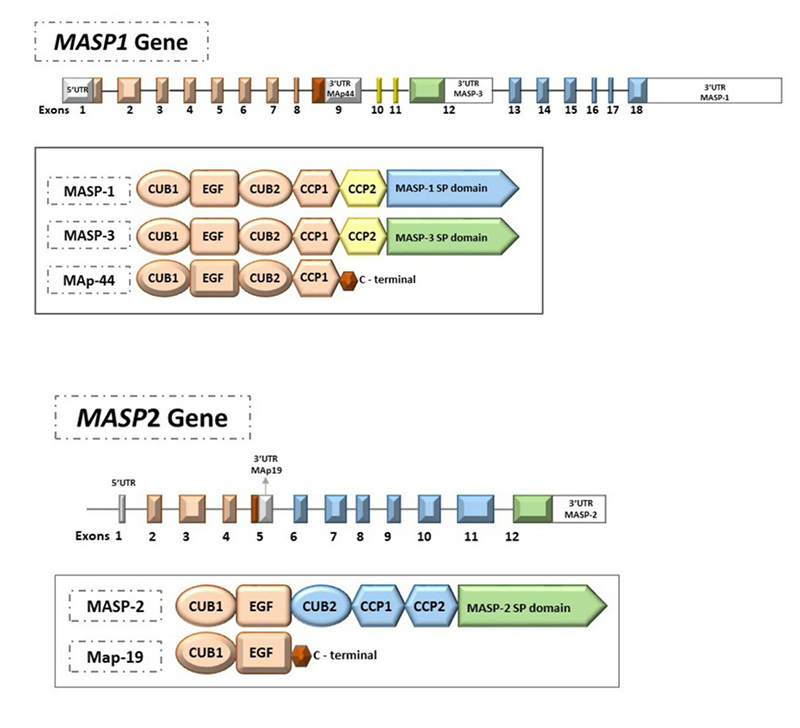
*MASP1* and **MASP2** genes. The *MASP1* gene (ENSG00000127241) generates three proteins by alternative splicing: MASP-1 (ENST00000337774.9), MASP-3 (ENST00000296280.11) and MAp44 (ENST00000169293.10). MASP-1 and MASP-3 share the regulatory domains (CUB1-EGF-CUB2-CCP1-CCP2), but differ in the serine protease domain (SP); MASP-1 SP is encoded by exons 13 to 18, whereas the MASP-3 SP is encoded only by exon 12. MAp44 lacks the SP domain but shares the first four domains with MASP-1 and MASP-3 (CUB1-EGF-CUB2-CCP1). This protein has no protease function. The *MASP2* gene (ENSG00000009724) encodes two proteins: MASP-2 (ENST00000400897.8) and MAp19 (ENST00000400898.3). MASP-2 and MAp19 share only two regulatory domains (CUB1-EGF). MASP-2 has a SP domain, but MAp19 lacks this domain. Genetic polymorphisms in these two genes can alter protein expression (see Tables 1 and 2), and many have already been associated with the most diverse disease models. Exons and introns are represented figuratively, not reflecting their actual size. The same colors represent the domains and their respective exons. Figure adapted from Boldt *et al*. (2016).

Genetic polymorphisms of the *MASP1* and *MASP2* genes are associated with low or high protein levels, modulating LP complement activation and susceptibility to diseases (Tables 1 and 2) (Stengaard-Pedersen *et al*., 2003; Sørensen *et al*., 2005; Thiel *et al*., 2007; Thiel *et al*., 2009; Rooryck *et al*., 2011; Ammitzbøll *et al*., 2013; Boldt *et al*., 2013; Boldt *et al*., 2016a, b; Krogh *et al*., 2017). The *MASP1* gene (3q27.3) encodes at least three proteins*:* MASP-1, MASP-3 and mannan-binding lectin-associated protein of 44 KDa (MAp44, also known as MAP-1) (Degn *et al*., 2009, 2010). The *MASP2* gene (1p36.22) encodes MASP-2 and mannan-binding lectin associated protein of 19 kDa (MAp19, also known as sMAP) (Stover *et al*., 1999). Their highest expression occurs in the liver, except MAp44, which is almost exclusively expressed in the heart (Degn *et al*., 2009) (Figure 2).

Whereas MASP-1 and MASP-2 activate the LP (Megyeri *et al*., 2013), MASP-3 cleaves pro-factor D into factor D and activates the AP. It also competes for the same binding sites on PRRs and may thus inhibit LP initiation (Dobó *et al*., 2016; Garred *et al*., 2016). On the other hand, MAp44 and MAp19 are truncated proteins that do not have the serine protease domain. MAp44 displaces MASPs from the collagenous stalks of the PRRs and blocks LP activation (Degn *et al*., 2009), while the function of MAp19 is still unclear, but assumed to be regulatory (Iwaki *et al*., 2006).

### MASPs and the coagulation cascades

MASPs have key roles in the interaction of the complement system, the coagulation cascade, and fibrinolysis, generating pro-inflammatory and/or pro-thrombotic responses that have not yet been fully elucidated (Hess *et al*., 2012; Dobó *et al*., 2014, 2018; Jenny *et al*., 2019). These components play a critical role in the physiology of comorbidities already associated with the worst prognosis of COVID-19, such as cardiovascular and cerebrovascular diseases, diabetes, metabolic syndrome and disseminated intravascular coagulation (DIC) associated with sepsis (Hess *et al*., 2012; Dobó *et al*., 2014; Jenny *et al*., 2019, Magro *et al*., 2020).

**Figure 3 -:**
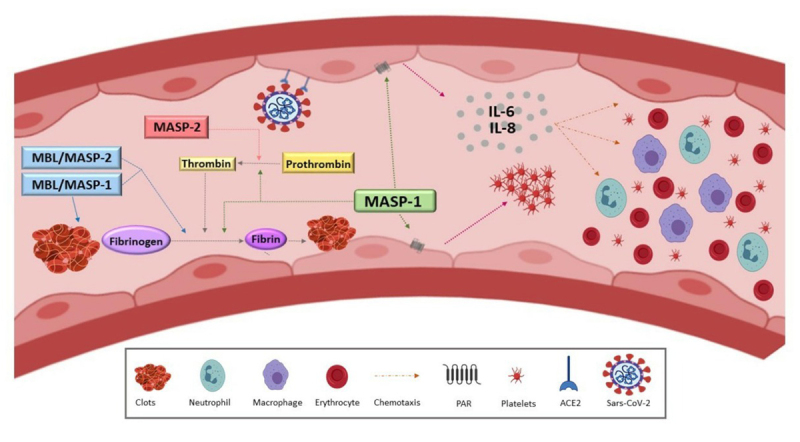
MASPs in the coagulation cascade during COVID-19. The participation of MASPs in coagulation contributes significantly to the formation of clots, and cleavage of intermediate components of the cascade. On the right side of the figure, MASP-1 activates endothelial cells through the protease-activated receptor (PAR) 4, 2 and 1. This activation has a characteristic response profile, and different from that stimulated via thrombin: activated endothelial cells start secretin pro-inflammatory cytokines and interleukins 6 and 8 (IL-6 and IL-8). Platelet aggregation also occurs. IL-6 and IL-8 contribute to the chemotaxis of neutrophils and macrophages. On the left, complexes of MASPs and MBL / MASPs participate in the coagulation cascade at different points. Like the factor Xa (FXa) of the coagulation cascade, MASP-2 cleaves prothrombin to thrombin l. MASP-1 participates in the cleavage of prothrombin to thrombin, and the cleavage of fibrinogen to fibrin. MASP-1 cleaves prothrombin in three specific sites: R393, R155, and R271. The sites R155 and R271 are shared with thrombin and factor FXa, respectively, whereas R393 is exclusive of MASP-1. MBL/MASPs complexes form clots directly, without co-participation of other components of coagulation (Gulla *et al*., 2010). These complexes cleave fibrinogen to fibrin, contributing to formation of clots in the coagulation cascade. MASP-1 has an independent dual role in the coagulation cascade and innate immunity. Its important contribution to the coagulation cascade and the time/structure of the clots can contribute to direct the pathophysiology of COVID-19 to severe conditions.

MASP-1 is the most abundant MASP in human serum. It has a promiscuous catalytic site, being the oldest protease of the CS (Endo *et al*., 1998; Degn and Thiel, 2013). The configuration of the MASP-1 serine protease domain (SP) differs from the C1r and C1s serine proteases and from other MASPs, being related to thrombin (Degn and Thiel, 2013; Dobó *et al*., 2014, 2018; Jenny *et al*., 2015a, b, 2019). In fact, MASP-1 substrates are not restricted to CS components, cleaving substrates as factor XIII (FXIII), quininogen, protease-activated receptor 4 (PAR4) in endothelial cells, prothrombin and the thrombin-activating fibrinolysis inhibiting antifibrinolytic factor (TAFI) (Krarup *et al*., 2007, 2008; Megyeri *et al*., 2009b; Dobó *et al*., 2014, 2018; Jenny *et al*., 2018, 2019). MASP-1 complexed with ficolins/MBL is directly associated with the formation of clots whose fibrin structure differs from common thrombin-mediated fibrinogen cleavage (Gulla *et al*., 2010; Takahashi *et al*., 2011; Hess *et al*., 2012). It acts in the later stage of clotting, accelerating the clotting process and clot formation in whole blood and platelet-poor plasma - physiological conditions that can be found in many pathologies and infectious diseases, including COVID-19 (Jenny *et al*., 2015b, 2019). MASP-1 and thrombin also activate endothelial cells in different ways, preferentially through PAR-4 and PAR-1, respectively (Megyeri *et al*., 2009a). Through PAR-4 activation, MASP-1 generates a specific pro-inflammatory immune response that promotes neutrophil chemotaxis and stimulates the production of IL-6 and IL-8, also attracting T cells in a severe infection (Dobó *et al*., 2014, 2018). A response very similar to the one described above has been reported in critically ill patients of COVID-19, however, the origin of this response remains unknown. Likewise, MASP-2 and factor Xa cleave prothrombin to thrombin, but the efficiency of MASP-2 seems to be lower (Krarup *et al*., 2007) (Figure 3).

The overactivation of coagulation in the initial phase of COVID-19, generating high levels of D-dimers, thrombocytopenia, and moderate prothrombin time prolongation (Thomson *et al*., 2020), seems to be caused by the combined action of MASP-1/MASP-2 and thrombin. The action of MASP-1 at a later stage may accelerate the time of clot formation, via prothrombin, in platelet-poor whole blood, converging in the formation of D-dimers and clots. Taken together, these data could contribute to explain the high levels of D-dimer and DIC observed in COVID-19 already in the initial phase, associated with new coronavirus pneumonia (NCP) (Jenny *et al*., 2015a; Tang *et al*., 2020b).

**Figure 4 -:**
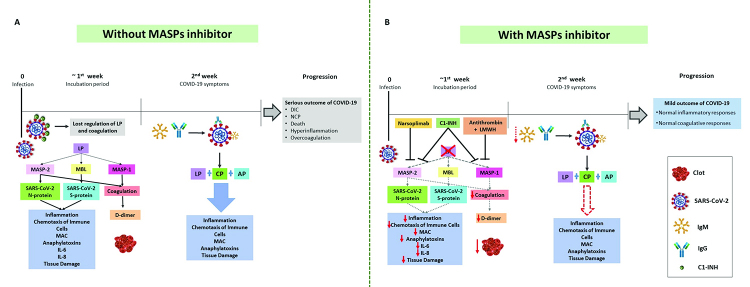
Promising therapeutic targets. (A) The clinical symptoms of COVID-19 appear after the first week of infection. In this first week, MASP-2 recognizes the N-SARS-CoV-2 protein and MBL recognizes S-SARS-CoV-2, leading to lectin pathway (LP) activation. Deficiency of C1-INH, proposed as a direct consequence of SARS-CoV-2 infection, leads to loss of physiological control of LP and coagulation, causing hyperinflammation and overcoagulation. During the second week, the symptoms start with activation of the classical pathway (CP) through the production of IgG and IgM antibodies, amplified by the alternative pathway (AP). At this stage of the disease, uncontrolled complement activation of all three pathways contribute to worsen the prognosis of COVID-19. (B) Drugs that inhibit the LP of complement can be considered as promising therapeutic agents against COVID-19. Those that inhibit MASP-1 and/or MASP-2 (Narsoplimab, C1-INH and antithrombin in the presence of low-molecular weight heparin - LMWH) are predicted to block both LP and coagulation, redirecting the clinical course of COVID-19 towards a better prognosis. This may also occur by reducing excessive antibody production, due to lower viral opsonization, phagocytosis and antigen presentation to B lymphocytes. AP - alternative pathway of complement; DIC - disseminated intravascular coagulation; C1-INH - C1 inhibitor; CP - classical pathway of complement; Ig - immunoglobulins; IL - interleukin; LP - lectin pathway of complement; MAC - membrane attack complex; MASP - mannose-binding lectin associated serine protease; NPC - new coronavirus pneumonia. Traced lines - weakened or blocked activation/ production. Solid lines - activation or blockage. Down-pointing arrows - lower production.

MASP-1 and MASP-2 have a catalytic role for both prothrombin and fibrinogen, therefore, they have the ability not only to activate the coagulation cascade, but also to contribute along with thrombin to thrombotic complications (Berlin *et al*., 2020; Jodele and Köhl, 2020) (Figure 4). Anticoagulant therapies with low-molecular weight heparin (LMWH) and antithrombin have shown a major impact on treatment outcome and prevention of DIC associated with COVID-19 (Fogarty *et al*., 2020; Lin *et al*., 2020; Ranucci *et al*., 2020), reducing mortality (Tang *et al*., 2020a). In the presence of LMWH, antithrombin and C1-INH are effective MASP-1 inhibitors (Dobó *et al*., 2009; Paréj *et al*., 2013), blocking both the LP and the cascade of coagulation. C1-INH also blocks MASP-2 (Kajdácsi *et al*., 2020). Interestingly, a deficiency of C1-INH is proposed as a direct consequence of SARS-CoV-2 infection, leading to loss of physiological control of LP and coagulation (Thomson *et al*., 2020). The MASP-2 inhibitor Narsoplimab has similar effects (Rambaldi *et al*., 2020). Interestingly, MASP-2 inhibitors improved endothelial cell damage in patients with thrombotic microangiopathy, who also presented high MASP-2 levels (Elhadad *et al*., 2020).

### Lectin pathway and coronavirus infection

**Figure 5 -:**
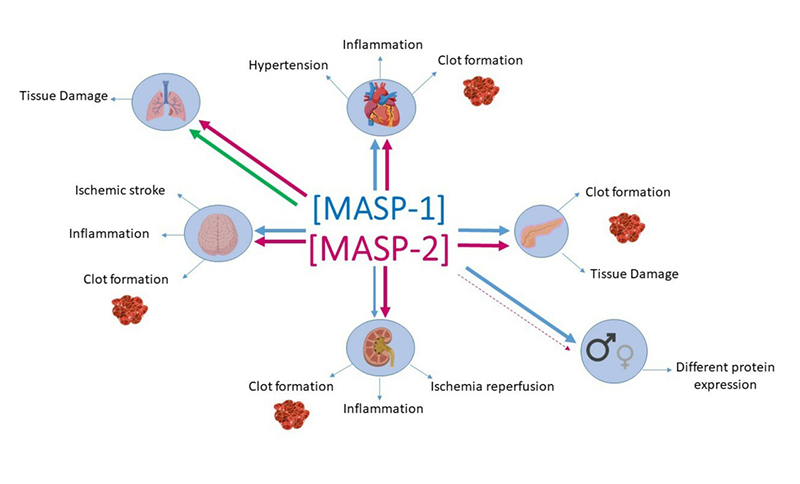
Relationship between MASPs and COVID-19 comorbidities. Changes in MASPs levels have already been associated with COVID-19 risk factors, such as sex, diabetes, kidney, cardiovascular, cerebrovascular, and chronic obstructive pulmonary disease (COPD). Genetic polymorphisms in the *MASP1* and *MASP2* genes influence the expression levels of these proteins. *COPD*: high levels in plasma and increased detection in situ, after MASP-2 was associated with risk (thick pink arrow), MASP-1 has not yet been associated/investigated with COPD, but the evidence leads us to suggest that MASP-1 function is also a risk factor to these patients (green arrow). *Arterial Hypertension and Cardiovascular Disease (CVD):* high levels of MASP-1 and MASP-2 have been associated with risk. The participation of MASP-1 in the coagulation cascade contributes to the formation of clots, worsening CVD. *Chronic kidney disease (CKD):* high levels of MASP-2 are associated with ischemia-reperfusion injury (IR), and MASP-1 seems to play crucial roles in chronic kidney disease, as well as its associated symptoms and conditions, such as IR and kidney transplantation. *Diabetes (DM):* the natural hyperglycemic status, uncontrolled activation of the lectin pathway, with elevated MASPs concentration in DM patients, might partially explain why those individuals belong to the group with higher incidence and severity of COVID-19. *Immunodeficiency and Immunosuppression:* MASPs levels have been associated with the condition and outcome of HIV and immunosuppressed patients, who also can be considered more vulnerable to COVID-19 infection. Apparently, MASP-1 and MASP-2 levels were not associated with susceptibility to infections in patients with immunosuppression, for this reason, the topic is not represented in this figure. *Cerebrovascular diseases:* MASP-1 and MASP-2 show increased proteolytic activity in the ischemic stroke, inflammation, and clots formation. *Sex:* About two-thirds of COVID-19 patients are men, who also have a higher expression of MASP-1 and lower expression of MASP-2. The disseminated intravascular coagulation observed in severe COVID-19 are representations such as the formation of clots in the organs where this activity has already been observed. The thickness of the arrows indicates MASP expressions in each comorbidity. The blue arrows correspond to MASP-1 and the pink arrows to MASP-2. The dashed pink line means a decrease in MASP-2 expression.

The LP is most probably the first to get activated during the incubation stage of SARS-CoV-2 infection, through the recognition of virus proteins by MBL and MASP-2 (Gao *et al*., 2020b; Magro *et al*., 2020; Stahel and Barnum, 2020). MBL also binds SARS-CoV-1 S-protein through a N-linked glycosylation site, inhibiting viral infection in susceptible cell lines (Ip *et al*., 2005; Zhou *et al*., 2010; Gao *et al*., 2020b; Jodele and Köhl, 2020). Furthermore, the nucleocapsid (N) protein of SARS-CoV-1, MERS-CoV and SARS-CoV-2 bind to MASP-2, inducing complement hyperactivation (Jodele and Köhl, 2020). Interestingly, the SARS-CoV-2 N-protein does also interact with MAp19 (Liu *et al*., 2009). The CP and AP are activated a few days later after production of C3b and antibodies, respectively, but antibody production may be compromised due to lymphocytopenia (Berlin *et al*., 2020; Stahel and Barnum, 2020; Matricardi *et al*., 2020) (Figures 1 and 5). In accordance with these findings, MASP-2, as well as MBL, C4a, C4, and C5b-9, accumulate in lung tissue of COVID-19 patients (Gao *et al*., 2020b). The lung microvasculature of severe COVID-19 cases also present deposits of MASP-2, C5b-9 and C4d (Magro *et al*., 2020). Thus, the LP seems to drive the primary immune response against SARS-CoV-2, but on the other hand, its chronic activation contributes to worsen COVID-19 symptoms (Eriksson *et al*., 2019; Gao *et al*., 2020b; Magro *et al*., 2020).

### Polymorphisms and protein levels of MASPs and coronavirus infection

*MASP1* and *MASP2* polymorphisms are associated with protein levels and with the susceptibility to different viral infections (reviewed by Beltrame *et al*., 2015) (Tables 1 and 2). *MASP2* polymorphisms rs2273346 (p.Val377Ala) and rs12711521 (p.Asp371Tyr) were investigated regarding susceptibility to SARS-CoV-1 infection, but no association was found (Wang *et al*., 2009). The absence of association may be due to the lack of investigation of common *MASP2* intronic regulatory variants, which present a much higher associated effect on MASP-2/MAp19 levels (rs2273344, rs9430347 and rs17409276) (Boldt *et al*., 2013) (Table 2). Thus, the role of the *MASP2* gene polymorphisms in SARS-CoV-2, such as of other CS genes, remains unclear.

To date, neither MASP-1 concentrations nor *MASP1* polymorphisms have been investigated in the inflammatory and/or hematological processes of COVID-19. Unlike other CS components already associated with COVID-19, routine analysis of hospitalized patients for the presence/concentration of MASP-1 has not been carried out (Magro *et al*., 2020). However, since MASP-2 strictly depends on MASP-1 for LP activation, and given its important role linking the LP to the coagulation cascade and association with many of the factors/comorbidities increasing the risk for severe COVID-19 disease (see below), we strongly suggest both as major candidates for further functional and genetic association studies in coronaviral diseases (polymorphisms listed on Tables 1 and 2). Furthermore, complement-induced lung injury may be attenuated by blocking the N-protein/MASP-2 interaction and/or by complement suppression itself, as suggested by data in vitro and in vivo (Gao *et al*., 2020b). In this context, coding polymorphisms may hinder recognition by monoclonal antibodies. This has been described for anti-C5 drugs (Nishimura *et al*., 2014), and may be theoretically possible for any coding polymorphism in the *MASP1* and *MASP2* genes, as well. In the case of the MASP-2 inhibitor already being used in compassionate treatment (Rambaldi *et al*., 2020), their impact will be important for *MASP2* p.Y371D (rs12711521), whose frequency of homozygotes reaches 25% in major human groups, but will be negligible for rare variants and those already reducing or abrogating protein function, as p.D120G (rs72550870) and p.R439H (rs12085877), and perhaps p.P126L (rs56392418), which may cooccur with p.439H in the *1C2-l [*AG*] haplotype (Boldt *et al*., 2013) and p.V377A (rs2273346) (see populational frequencies in Table 2).

MASPs are actually predicted to get engaged with the virus as soon as it enters peripheral circulation, either by directly attaching to it (MASP-2), or as homodimers connected to PRRs that recognize sugar and acetylated moieties at the viral surface (Gao *et al*., 2020b; Jodele and Köhl, 2020; Świerzko and Cedzyński, 2020). They may be explored as biomarkers for disease progression. Decreasing MASP levels may predict increasing disease severity, due to the enrollment of MASPs in the coagulation and complement cascades, as well as in phagocytic engulfment of opsonized particles, exacerbating inflammation. In fact, lower MASP-2, MASP-3 and MAp44 levels occur in leprosy and lepromatous disease, probably due to its consumption in the inflammatory response. On the other hand, *MASP1* haplotypes associated with lower MASP-1 and higher MASP-3 levels at baseline were associated with leprosy resistance, probably by decreasing mycobacteria infection, which relies on successful complement-driven opsonization and phagocytosis (Boldt *et al*., 2013; Weinschutz Mendes *et al*., 2020). Thus, the investigation of MASP levels in COVID-19 is also highly relevant, especially in the context of comorbidities.

### Complement-targeted therapies

Due to the high pleiotropy of genes encoding complement components, it is impossible to predict precisely the effect of inhibiting one of its molecules. This challenge however, has been partly overcome with animal models. In the case of mice infected with mouse adapted SARS-CoV-1, weight loss and respiratory dysfunction was reduced by C3 complement knock-out, although viral loads did not differ in the lungs of wild-type and C3 knock-out mice (Gralinski *et al*., 2018). Decreased disease severity was also found after complement inhibition with an anti-C5a reagent in the MERS-CoV human DPP4 transgenic mouse model (Jiang *et al*., 2018). Interestingly, both C3 (compstatin-based C3-targeted drug candidate AMY-101) and C5 (Eculizumab) inhibitors enhanced lung function concomitant with a steep decline in C-reactive protein and IL-6 levels. However, C3 inhibition also reduced C3a and sC5b-9 generation and prevented factor B (of the AP) consumption (Mastellos *et al*., 2020). It may also disrupt tissue factor expression in neutrophils (Skendros *et al*., 2020). This broader C3inh-dependent therapeutic control was also reflected by lower neutrophil counts and less NET release, stronger lymphocyte recovery and faster serum lactate dehydrogenase (LDH) decline (Mastellos *et al*., 2020). The usage of C3 and C5 inhibitors is currently being tested in six different clinical trials registered in the ClinicalTrials.gov database and presents a high therapeutic potential (Lo *et al*., 2020).

Unlike these, MASP-2 blockage is not expected to interrupt CP (Figure 4). Even so, usage of an anti-MASP-2 human monoclonal antibody (Narsoplimab) on six Italian patients with COVID-19 acute respiratory distress syndrome (ARDS) was highly effective, without any measurable colateral effect, achieving fast and sustained reduction of circulating endothelial cells (a marker of vascular damage) and concomitant reduction of serum IL-6, IL-8, C-reactive protein (CRP) and LDH (Rambaldi *et al*., 2020). In comparison, C5 and C3 inhibition (e.g. with Eculizumab) are effective in blocking formation of the C5 convertase, the C5a anaphylatoxin and the membrane attack complex, which are terminal steps achieved by all complement initiating pathways (Laurence *et al*., 2020; Mastellos *et al*., 2020). However, both would not necessarily affect the coagulation cascade. In the case of COVID-19, blocking LP through a MASP inhibitor may suffice to inhibit hyperinflammation and microcoagulation events, because (1) this pathway appears to be the first to get activated in contact with the virus, (2) MASPs activate key arms of the coagulation cascade and (3) MASP2 can recognize SARS-Cov-2 viral N protein. In fact, although all three strategies target CS components with the potential to control hyperinflammation, only the MASP inhibitors have the ability to control hyperinflammation, clotting and viral recognition at once (Figure 4). In fact, Narsoplimab blocks MASP-2-mediated activation of kallikrein and factor XII and consequent microvascular thrombosis, benefitting patients with massive pulmonar thrombotic events. It cannot block extracellular matrix-related, factor VII-driven, coagulation and does not prolong bleeding time nor affect prothrombin or activated partial thromboplastin times (Demopulos *et al*., 2020; Omeros Corporation, 2013a, b; 2019). Furthermore, the broader action of C5 inhibition on complement increases susceptibility to opportunistic pathogens as *Neisseria meningitidis*, even in vaccinated individuals (Langereis *et al*., 2020). Thus, combining complement-inhibiting strategies would expose the patients to unnecessary risks, and unilateral inhibition of just LP, which seems to be the major complement pathway in the disease, would preserve most needed complement-provided bridges to a well-regulated adaptive immune response. Caution must be nevertheless observed at this point, since we partly build our assumptions on basic science and partly on pioneering clinical interventions. More translational studies are undoubtedly needed to arrive at a better therapeutic strategy in patients with COVID-19.

### MASPs and COVID-19 risk factors

Age and sex

Age and sex are well-known aspects that could impact the quality of the immune response (Giefing-Kröll *et al*., 2015; Gaya da Costa *et al*., 2018). A study performed with Chinese patients with COVID-19 revealed that 0.9% of the patients were younger than 15 years of age, and 15.1% had more than 65 years (Guan *et al*., 2020). Impressively, the median age of the patients with severe cases was higher, and more than half of patients (58.3%) who developed severe symptoms were over 50 years old. Just 12.9% of those without severe disease were more than 65 years old (Guan *et al*., 2020), and symptoms presented a slow course in children compared to infected adults (Lu *et al*., 2020). In a Brazilian study with 510 COVID-19 patients, the average age was 40 years. Only 0.6% of patients were under 11 years old, and 6.5% were over 65 years old (Teich *et al*., 2020).

Although neonatal innate immunity is focused on protection against extracellular pathogens instead of intracellular bacteria and viruses (Prendergast *et al*., 2012), elderly usually have just 1% of new T cell production compared to a child (Vallejo, 2006; Chinn *et al*., 2012). Thus, older adults are more susceptible to infections, mainly due to the reduction of efficiency and coordination of immune response, added to comorbid conditions, and the simultaneous use of multiple drugs (Weng, 2006; Shaw *et al*., 2013; Nikolich-Zugich *et al*., 2020). Since COVID-19 has been associated with coagulation (Zhang *et al*., 2020e), better condition of blood vessels in children could be a protection factor against the infection (Cyranoski, 2020). Nevertheless, age does not seem to affect the levels of LP components, in contrast with the circadian cycle, which plays a prominent role for MAp19, ficolin-3 and ficolin-1 levels (Troldborg *et al*., 2017). Pronounced age-related differences appear to happen only in the first year of life, with MASP-2 levels being considerably lower in preterm and term neonates, than in adults (Sallenbach *et al*., 2011).

COVID-19 infection is more common in males than in females (Rodriguez-Morales *et al*., 2020; Zhu *et al*., 2020; Teich *et al*., 2020), representing 58% (Guan *et al*., 2020) to 67% of the cases in Chinese population (Chen *et al*., 2020a). Similarly, in a Brazilian study, 59.6% were male (Teich *et al*., 2020). The hormone profile seems to be the key to these differences (Fairweather *et al*., 2008; Giefing-Kröll *et al*., 2015; Gaya da Costa *et al*., 2018). Hormone receptors bind to estrogens and androgens, which may impact the immune response (Fairweather *et al*., 2008). X chromosome inactivation (Schurz *et al*., 2019) and unequal expression of X-linked genes may also partly explain these differences (Ghorai and Ghosh, 2014; Klein and Flanagan, 2016). In general, the female immune response presents a better performance on vaccines and viral responses, at the cost of increased susceptibility to inflammatory and autoimmune diseases (Klein and Flanagan, 2016; Roved *et al*., 2017). In the context of the CS, high levels of serum MBL, ficolin-3, and MASP-1 and lower levels of MASP-2 (Troldborg *et al*., 2017; Gaya da Costa *et al*., 2018) were found in men compared to women, but MASP-3, MAp19 and MAp44 serum levels did not differ between sexes (Gaya da Costa *et al*., 2018) (Figure 5).

Arterial hypertension and cardiovascular disease (CVD)

Arterial hypertension is a multifactorial health problem, characterized by high blood pressure, which affects around 1.13 billion people (WHO, 2020b) and causes 13% of deaths worldwide (Chowdhury *et al*., 2020). It is a primary risk factor for premature cardiovascular diseases (CVD) and mortality. In Brazil, the prevalence was about 24.3% in 2017, affecting 61% of adults above 65 years of age (Brasil - Ministério da Saúde, 2017). Age, sex, body mass, as well as environmental and genetic factors, contribute to rising blood pressure (Sabbatini and Kararigas, 2020).

Hypertension might be one of the most frequent comorbid diseases in patients with COVID-19 (Gao *et al*., 2020a; Guan *et al*., 2020; Hu *et al*., 2020). Gao *et al*. (2020a) showed that patients with hypertension have a two-fold increase in the mortality risk (adjusted hazard ratio [HR] = 2.12, 95%CI = 1.17-3.82, p = 0.013) (Gao *et al*., 2020a).

Interestingly, SARS-CoV-2 enters human cells binding the Angiotensin-converting enzyme 2 (ACE2) (Zhou *et al*., 2020a). Hypertension is usually controlled with ACE inhibitors and angiotensin II type-I receptor blockers (ARBs), drugs that up-regulate ACE2, probably increasing opportunities for viral infection (Fang *et al*., 2020). Being expressed at blood vessels, ACE2 is part of the cardiovascular system. Thus, viral interaction can increase myocardial dysfunctions in cases with prior cardiovascular issues (Askin *et al*., 2020). Additionally, ACE2 is also expressed in the lung, intestinal epithelium, vascular endothelium, and kidneys, which is one of the causes of multiple organ failure in SARS-CoV-2 infection (Tikellis and Thomas, 2012; Zhang *et al*., 2020a). Higher levels of pro-inflammatory cytokines and coagulation activation factors indicate that inflammatory responses and the prothrombotic state are involved in COVID-19 symptoms (Chen *et al*., 2020a). As mentioned before, MASP-1 and MASP-2 play an important role in the coagulation system, activating several coagulation factors (Figure 3) (Kozarcanin *et al*., 2016). Indeed, MASP-1 levels are higher in myocardium infarction patients, as well as MASP-2 in coronary artery disease patients (Frauenknecht *et al*., 2013). MAp44 showed cardioprotective and antiatherothrombotic effects in two murine models, preserving cardiac function (Pavlov *et al*., 2012). Unexpectedly, in humans its levels correlated positively with cardiovascular risk factors, as dyslipidaemia, obesity and hypertension (Frauenknecht *et al*., 2013). Furthermore, *Masp2* knockout mice presented smaller infarct injuries resulting from postischemic myocardial ischemia reperfusion (IR). Interestingly, the injuries in wild-type mice depend on Masp-1, Masp-2, C2 and C3 (but not C4), and the injection of a murine-specific Masp-2 inhibitor greatly reduced the size of IR-induced gastrointestinal injuries (Schwaeble *et al*., 2011). Therefore, pharmacological inhibition of MASPs may reduce the risk to cardiovascular disease and improve the treatment of COVID-19 disease (Figure 5).

Chronic kidney disease (CKD)

Chronic kidney disease (CKD) is characterized as a reduction of glomerular filtration rate and/or increased urinary albumin excretion (Jha *et al*., 2013), and affects 10-15% of adults worldwide (Zhang *et al*., 2020d). In Brazil, the prevalence of CKD is approximately 500 per million individuals (Jha *et al*., 2013). Diabetes and hypertension are the leading causes of CKD in many countries (Jha *et al*., 2013). However, genetic and environmental factors, the prevalence of infectious diseases, aging, and access to health care, can alter CKD epidemiology (De Nicola *et al*., 2015). Complications include cardiovascular events, kidney-disease progression, acute kidney injury, cognitive decline, and anaemia (Jha *et al*., 2013).

Kidney disease is also a major complication of COVID-19 and a significant risk factor of death (Cheng *et al*., 2020b). It was previously reported that 6.7% of patients with SARS-CoV-1 developed acute kidney injury (Chu *et al*., 2005). Furthermore, the incidence of in-hospital death was higher in patients with elevated baseline serum creatinine levels (33.7%), in comparison to those with normal baseline serum creatinine levels (13.2%) (Cheng *et al*., 2020b). Cheng *et al*. (2020b) investigated 701 patients with COVID-19 from Wuhan, China. Of this total, 2.0% reported chronic kidney disease as comorbidity, thus already presenting a proinflammatory state with functional defects in innate and adaptive immune cell populations (Betjes, 2013).

LP-mediated complement activation might have a negative impact in the pathophysiology of CKD, because of its involvement in IR, transplant immunity, and in the coagulation cascade (Takahashi *et al*., 2011). In these settings, the inactivation of MASP-1 and MASP-2 could prove beneficial. Asgari *et al*., (2014) indicated that MASP-2 presents a critical role for renal injury. In a *Masp2* knockout mouse transplant model, its absence protected the transplanted kidney from IR injury, with less C3 deposition, but in a C4-bypass independent mode (Asgari *et al*., 2014). Blocking Masp-2 reduces the harmful effects of proteinuria, by inactivating LP in proximal tubular cells of wild-type mice. This was as effective as in *Masp2* knockout mice (Alghadban *et al*., 2019) (Figure 5). Yet low levels of MAp44 at the time of kidney transplantation were associated with increased mortality caused by infectious diseases in the recipients, after nearly 14 years of transplantation (Smedbråten *et al*., 2016). Therefore, MASP-1/-2 and MAp44 seem to play crucial roles in chronic kidney disease, as well as its associated symptoms and conditions, such as IR and kidney transplantation.

Diabetes

Long before the pandemic caused by SARS-CoV-2, the world has been experiencing the silent pandemic of diabetes mellitus (DM) (Santos *et al*., 2019; reviewed by Toniolo *et al*., 2019). In Brazil, the prevalence of the main risk factors for DM, elevated body mass index and obesity, are 55.7% and 19.8% respectively. Currently, DM occupies the third position as the most common factor leading to death. Its prevalence is about 8% in the Brazilian population (16.8 million people) and over 425 million people worldwide (Marinho *et al*., 2018; Saeedi *et al*., 2019). Along with a wide range of disease-associated complications, patients with DM present a variety of metabolic, vascular, and immune abnormalities which, combined with their inflammatory status, affect their response to pathogens (Mellbin *et al*., 2012; Kulcsar *et al*., 2019).

Previous viral epidemics, like SARS-CoV-1 and MERS-CoV have shown that DM and uncontrolled glycaemia were among the predictive factors of severity and death in infected patients (Hussain *et al*., 2020). Some immune features observed in the pathogenesis of those viral infections in DM patients were related to dysfunction of several cellular immune components, but the humoral innate component, including the CS, have been poorly explored in DM patients, during those epidemics (Kulcsar *et al*., 2019; Singh *et al*., 2020). As observed in these respiratory viral infections, preexisting DM is associated with higher incidence, severity and mortality in COVID-19 patients (reviewed by Chen *et al*., 2020c; Wang *et al*., 2020b; Wu *et al*., 2020). Besides the common immune mechanisms, new predisposing factors might be responsible for the increased severity and even risk for SARS-CoV-2 infection in individuals with DM, as increased levels of IL-6, impaired T-cell function and higher expression of furin and ACE-2 (reviewed by Cristelo *et al*., 2020; Singh *et al*., 2020). This last receptor is probably up-regulated due to the common treatment of type 1 and type 2 diabetes with ACE inhibitors and ARBs, also used for hypertension (Fang *et al*., 2020). The dysfunction of complement activation in DM may have a major participation in inflammatory processes, endothelial damage, activation of blood coagulation and vascular complications, all common adverse outcomes of DM itself and of COVID-19 (reviewed by Ghosh *et al*., 2015; Huang *et al*., 2019).

The constant activation of the complement system, in the DM context, might be due to the production of advanced glycation end-products (AGE) and generation of neoepitopes by the hyperglycaemic environment and their interaction with MBL and MASPs. In fact, MBL binding to AGE induce conformational changes that activate MASP-1, and subsequently, MASP-2 (Jenny *et al*., 2015a; Pelletier *et al*., 2019). In prediabetes, type 1 and type 2 DM, plasma levels of MBL, MASP-1 and MASP-2 are elevated, and animal models have shown that MASP-1 levels rise just before the onset of DM symptoms (Jenny *et al*., 2015a; Krogh *et al*., 2017; Huang *et al*., 2019; Huth *et al*., 2019). Genetic association with protein levels was studied only in type 2 DM patients and relative to *MASP1*, of which five SNPs (rs874603, rs72549254, rs3774275, rs67143992, rs850312) were associated with plasma levels of MASP-1, MASP-3 and MAp44, but only MASP-1 levels were associated with the disease itself (however, the possible additive effect of the variants in a haplotype context, was not investigated) (Table 1) (Krogh *et al*., 2017). The link between hyperglycaemia and complement activation might also play an important role in the pathogenesis of SAR-CoV-2, since the control of blood glycemia seems to reduce death rates in DM infected patients (Hussain *et al*., 2020; reviewed by Cristelo *et al*., 2020; Fadini *et al*., 2020; Singh *et al*., 2020) (Figure 5).

**Table 1 -:** *MASP1* gene polymorphisms associated with MASP-1, MASP-3, and MAp44 serum levels.

dbSNP	Allele	Gene and protein region	Amino acid position	AF Brazilian¹ %	AF AFR %	AF EUR %	AF EAS %	Serum levels*
rs190590338	G>**A**	Promoter - n.a	n.a.	n.o.	0	<1	0	Higher MASP-1 levels in G/A (Ammitzbøll *et al*., 2013)
**rs7625133**	A>**C**	Promoter - n.a	n.a.	n.o.	2.4	6.4	<1	Lower MAp44 levels in A/C and C/C (Ammitzbøll *et al*., 2013)
**rs35089177**	**T>A**	Promoter - n.a	n.a.	n.o.	6.3	34.3	38	Lower MASP-1 and MAp44 levels in T/A and AA (Ammitzbøll *et al*., 2013)
rs75284004	A>**G**	Promoter - n.a	n.a.	n.o.	<1	4.1	<1	Lower MASP-3 levels in A/G (Ammitzbøll *et al*., 2013)
rs62292785	G>**A**	Promoter - n.a	n.a.	n.o.	4.8	5.6	18.8	Lower MASP-1 levels in G/A (Ammitzbøll *et al*., 2013)
**rs72549254**	G>**A**	Intron 1 - n.a	n.a.	13.3	21.9	13.9	11.7	Higher MASP-3 levels in A/G (Ammitzbøll *et al*., 2013) Lower MAp44 levels in A/G and A/A (Ammitzbøll *et al*., 2013) Lower MAp44 levels in G/G (Krogh *et al*., 2017)
rs710469	C>**T**	Intron 2 - n.a	n.a.	n.o.	35.4	50.5	51.1	Higher on-admission MASP-3 levels in critically ill children with T/T (Ammitzbøll *et al*., 2013)
**rs3774275**	A>**G**	Intron 8 - n.a	n.a.	18.5	2.1	30.8	41.8	Higher MASP-1, Map44 and lower MASP-3 levels in A/G and G/G (Ammitzbøll *et al*., 2013) Lower MASP-1 and MAp44 levels in A/A (Krogh *et al*., 2017)
rs113938200	C>**T**	Exon 9 - C-terminal MAp-44	p.Asn368Asp	<1	0	<1	0	Lower MAp44 levels in C/T (Ammitzbøll *et al*., 2013)
rs698090	**C>T**	Exon 9 - 3’UTR Map-44	n.a.	37.4	62.4	35.8	37.6	Higher MASP-1, MAp44 and lower MASP-3 levels in C/C (Ammitzbøll *et al*., 2013) Higher MAp44 levels in C/T (Ammitzbøll *et al*., 2013)
rs72549154	G>**T**	Exon 12 - SP MASP-3	p.Arg576Met	<1	15.1	2.5	<1	Lower MASP-1 levels in G/T (Ammitzbøll *et al*., 2013)
rs850312	G>**A**	Exon 12 - CCP2 MASP-3	p.Leu617Leu	27.2	2.6	34.8	20.3	Higher on-admission MASP-3 levels in critically ill children with A/A, A/G (Ammitzbøll *et al*., 2013) Lower MASP-3 and higher Map44 levels in G/G (Krogh *et al*., 2017)
rs67143992	G>**A**	Exon12 - 3’ UTR MASP-3	n.a.	12.9	1.4	16.9	4.5	Higher MASP-1, MAp44 and lower of MASP-3 levels in G/A (Ammitzbøll *et al*., 2013) Higher MAp44 and lower MASP-3 levels in A/A (Ammitzbøll *et al*., 2013) Lower MASP-3 levels in A/A (Krogh *et al*., 2017)
**rs874603**	**G>A**	3’ UTR MASP-3	n.a.	12.4	36.2	5.7	0.8	Higher MASP-3 levels in G/A (Krogh *et al*., 2017)

dbSNP: Single Nucleotide Polymorphism Database; n.o.: no data; n.a.: not applicable; AF: allele frequency of 1000 genomes project from AFR, EUR and EAS; AFR: African, EUR: European, and EAS: East Asian (data accessed on: https://www.ensembl.org/index.html); CCP: Complement Control Protein; SP: Serine Protease; UTR: untranslated. *compared to the homozygote state of the other allele. Alleles in bold are considered the minor alleles in the global population. Serum levels are related to this allele. SNPs in bold are considered expression quantitative trait loci (eQTLs), due to their association with mRNA expression. (http://www.gtexportal.org/home/eqtls/byGene?geneId=MASP1&tissueName=All) ¹data from ABraON (http://abraom.ib.usp.br/index.php). Table modified from Boldt *et al*., 2016a.

Chronic obstructive pulmonary disease (COPD)

Chronic obstructive pulmonary disease (COPD) affects the airway and other pulmonary structures, resulting in airflow limitations caused, predominantly, by exposure to noxious particles or gases over a long period (reviewed by Decramer *et al*., 2012). The worldwide prevalence of COPD is estimated to be 4.76% (328 million cases) (Vos *et al*., 2012). In São Paulo, the most populated city of Brazil, this prevalence reaches 15.8% (Platino and Rosa, 2005).

The exacerbations of COPD have been frequently associated with viral infections (Retamales *et al*., 2001; reviewed by Leung *et al*., 2017; reviewed by Zwaans *et al*., 2020). Patients with pre‐existing COPD infected by SARS-CoV-2 are 4.4 times more likely to develop severe COVID-19, resulting in worse outcomes (Zhao *et al*., 2020) and higher mortality rates (Alqahtani *et al*., 2020).

*MBL2* polymorphisms seem not to be associated with COPD per se. In the Danish population, genetic MBL deficiency was not considered a major risk factor for COPD (Dahl and Nordestgaard, 2009), and low MBL levels were not associated with COPD in Norwegian population (Eagan *et al*., 2010). Still, C3d deposition was detected in a set of 36 intensive care unit patients who died while on invasively mechanical ventilation, 12 of whom were diagnosed with ARDS (Beer *et al*., 2019). However, MBL levels were lower in bronchoalveolar lavage of COPD patients (Hodge *et al*., 2008). Furthermore, the deficiency-causing MBL2*B allele (rs1800450) was associated with 4.9 times increased odds of hospital admission for infection-induced COPD exacerbation (Yang *et al*., 2003). This was confirmed in another setting, where *B/B* homozygosity and the heterozygosity combined with the down-regulating promoter alleles *XA/B* were not only associated with MBL deficiency, but also with frequent infection exacerbation in COPD, and worse outcomes (Lin *et al*., 2011). Furthermore, the LP seems to be activated in COPD, both in plasma and in situ. MASP-2 levels and LP activation were higher in plasma, and MASP-2 was also detected at higher amounts in the airways and lung parenchyma of COPD patients, compared with healthy individuals (Serban *et al*., 2019). Thus, the role played by MASPs and their polymorphisms in this disease deserves further investigation, especially regarding its effect on severe COVID-19 infection (Tables 1 and 2) (Figure 5).

Secondary immunodeficiency and immunosuppression

In former human coronavirus outbreaks, immunosuppressed individuals were not more prone to infections or severe complications. This scenario apparently changed with COVID-19 (D’Antiga, 2020). Among the secondary immunodeficiency causes enhancing COVID-19 severity, acquired immunodeficiency syndrome (AIDS) can be considered the most globally widespread. By the end of 2018, 37.9 million people were infected by the human immunodeficiency virus (HIV) globally, with 770,000 deaths in this same year (WHO, 2020c). Between 1980 and 2019, 966.058 AIDS cases were detected in Brazil (Brasil - Ministério da Saúde, 2019). AIDS is a condition caused by HIV infection, resulting in immunosuppression as a consequence of selective depletion of helper/inducer T lymphocytes that express the CD4 receptor (Fauci, 1988). Lopinavir-boosted ritonavir, antiretroviral treatment (ART) used for HIV infections, was tested against MERS-CoV and SARS-CoV-1 in the past, usually reducing viral loads (Chan *et al*., 2015; Sheahan *et al*., 2020). Following the hypothesis that HIV protease inhibitors might have activity against the coronavirus protease (pre-print Chang *et al*., 2020; Costanzo *et al*., 2020), lopinavir-boosted ritonavir and darunavir-boosted cobicistat, which are booted-protease inhibitors (ART), seemed to help the recovery of SARS-CoV-2 patients (Blanco *et al*., 2020). Conversely, these drugs did not shorten duration of SARS-CoV-2 viral shedding in patients with mild pneumonia (Cheng *et al*., 2020a). Later, in more extensive and robust tests, these ART were not found to be effective against SARS-CoV-2 in vivo (Cao *et al*., 2020; Jones *et al*., 2020).

The interaction between gp120 glycosylation sites present in the HIV envelope and MBL/MASP complexes have been established in vitro (Saifuddin *et al*., 2000; Hart *et al*., 2003). These observations lead to believe that MBL/MASP complexes, and LP as a whole, act in early viral inhibition (reviewed by Ballegaard *et al*., 2014). However, in HIV pathogenesis, CS and its PRRs have two conflicting roles (Yu *et al*., 2010). In one hand, increased susceptibility to HIV infection was associated with low MASP-2 serum levels due to genetic polymorphisms, such as rs56392418 (Boldt *et al*., 2016a). (Table 2), and very low MBL levels were also observed in CDC stage II HIV infected patients (Prohászka *et al*., 1997). On the other hand, the complement element C3 binds to the virus, facilitating the interaction of HIV with CR3 and CR4 receptors of dendritic cells in vitro (Bajtay *et al*., 2004; Pruenster *et al*., 2005). Furthermore, MASP-2 lower levels/ structural deficiency associated with p.120G, p.126L, p.377A and p.439H (rs72550870, rs56392418, rs2273346 and rs12085877, respectively) presented a protective effect against AIDS, probably by suppressing inflammation (Boldt *et al*., 2016a) (Table 2).

**Table 2:** - *MASP2* gene polymorphisms associated with MASP-2 and MAp19 serum levels.

dbSNP	Allele	Gene and protein region	Amino acid position	AF Brazilian¹ %	AF AFR %	AF EUR %	AF EAS %	Serum levels*
**rs7548659**	**G>T**	Promoter - n.a	n.a.	29.3	82.8	21.9	26.1	high MASP-2 and low MAp19 concentration (Boldt *et al*., 2013)
rs61735600	C>**T**	Exon 3 - CUB1	p.R99Q	1.3	7.1	0	0	high MASP-2 concentration (Thiel *et al*., 2007)
rs72550870	T>**C**	Exon 3 - CUB1	p.D120G	1.4	<1	3.9	0	low MASP-2 and MAp19 concentration (Stengaard-Pedersen *et al*., 2003; Sørensen *et al*., 2005; Thiel *et al*., 2007; Boldt *et al*., 2013)
rs56392418	C>**T**	Exon 3 - CUB1	p.P126L	0.5	13.2	0	0	low MASP-2 concentration (Thiel *et al*., 2009)
rs2273343	T>**C**	Exon 4 - EGF	p.H155R	n.o.	0	0	2.7	low MASP-2 concentration (Thiel *et al*., 2009)
**rs2273344**	**C>T**	Intron 4 - n.a	n.a.	18.6	10.1	20.4	11.8	high MASP-2 and low MAp19 concentration (Boldt *et al*., 2013)
**rs9430347**	G>**A**	Intron 5 - n.a	n.a.	18.6	9.2	20.4	11.6	high MASP-2 and low MAp19 concentration (Boldt *et al*., 2013)
**rs17409276**	G>**A**	Intron 9 - n.a	n.a.	14.7	16.6	13.8	13.6	high MASP-2 and low MAp19 concentration (Boldt *et al*., 2013)
**rs12711521**	**C>A**	Exon 10 - CCP2	p.D371Y	22.9	82.9	18	33.4	high MASP-2 and low MAp19 concentration (Boldt *et al*., 2013)
rs2273346	A>**G**	Exon 10 - CCP2	p.V377A	3.4	17.1	2	19.8	low MASP-2 concentration (Thiel *et al*., 2007; Thiel *et al*., 2009)
rs12085877	G>**A**	Exon 12 - SP	p.R439H	0.1	10.4	0	0	low MASP-2 concentration (Thiel *et al*., 2009)
**rs1782455**	**G>A**	Exon 12 - SP	p.S493=	19.5	70.3	16	13.5	high MASP-2 and low MAp19 concentration (Boldt *et al*., 2013)

dbSNP: Single Nucleotide Polymorphism Database; n.o.: no data; n.a: not applicable; AF: allele frequency of 1000 genomes project from AFR, EUR and EAS; AFR: African, EUR: European, and EAS: East Asian (data accessed on: https://www.ensembl.org/index.html); CCP: Complement Control Protein; SP: Serine Protease, EGF: epidermal growth factor. *Homozygote of the allele effect. Alleles in bold are considered the minor AF in global population and the serum levels are related to this allele. SNPs in bold are considered expression quantitative trait loci (eQTLs), due to their association with mRNA expression (http://www.gtexportal.org/home/eqtls/byGene?geneId=MASP2&tissueName=All). ¹data from predominantly Euro-Brazilian healthy volunteers (Boldt *et al*., 2016a). Table modified from Boldt *et al*., 2016a.

Nevertheless, immunosuppressive medications for cancer therapy increase the risk of severe viral infections, due to is effects on humoral, cell-mediated immunity, and neutrophil function (Kaltsas and Sepkowitz, 2012). In cancer patients, these infections may also delay the treatment, causing unnecessary hospitalization (Al-Quteimat and Amer, 2020). Several reports indicate that cancer patients should be regarded as an extremely vulnerable group during the COVID-19 pandemic (Ueda *et al*., 2020; Gosain *et al*., 2020; Zhang *et al*., 2020c). In a nationwide cohort in China, COVID-19 patients with cancer showed a higher probability (39%) to be admitted to intensive unit care and decease, if compared to COVID-19 patients without cancer (8%) (Liang *et al*., 2020). Therefore, new guidelines to approach cancer care during the pandemic are being developed (Gosain *et al*., 2020). Whether or not immunosuppressive medication for cancer treatment should be interrupted during the pandemic, is still under debate (Al-Quteimat and Amer, 2020; Ueda *et al*., 2020).

Patients with hematological malignancies have an impaired immune system not only due to the disease, but also because of its treatment. This scenario may be worsened by the presence of genetic variants lowering the efficacy of LP activation. In accordance with this, the MASP-2 deficiency-causing p.D120G polymorphism (rs72550870) was frequently found in lymphoma patients who experienced bacteremia during hospitalization, whereas fully-functional p.D371Y (rs12711521) was associated with decreased risk of diffuse large B‐cell lymphoma (Table 2) (Hu *et al*., 2013; Świerzko *et al*., 2018). MASP-2 levels were similar between healthy controls and pre-treatment multiple myeloma and lymphoma patients (Świerzko *et al*., 2018). In another group of pediatric patients with hematologic malignancies, high MASP-2 levels were significantly associated with higher chance of event-free survival (Zehnder *et al*., 2009). Neutropenia occurs when patients with cancer have a temporary reduction in their neutrophil numbers caused by chemotherapy. Therefore, the risk of infection greatly increases in these patients (Patel and West, 2017). As expected, children going through chemotherapy and with MASP-2 deficiency have a two-fold increased risk of developing febrile neutropenia (Schlapbach *et al*., 2007). In conclusion, the characteristic “double-edge” association of MASP-2 (and probably also of MASP-1 and other LP components) with immunosuppression, infection and inflammation poises a difficult question regarding therapies directed to its inhibition, motivating further investigations in this field (Figure 5).

Cerebrovascular diseases

Cerebrovascular disease is a general term that includes different brain vascular disturbances, leading to the vast majority of deaths due to stroke (Truelsen *et al*., 2000). They have been reported as a comorbidity that increases the likelihood of a worse prognosis by up to 3.5 times in patients with COVID-19 (Aggarwal *et al*., 2020; Wang *et al*., 2020a). Worldwide, the mortality rate due to cerebrovascular diseases was 101 per 100,000 until 2015 (Wang *et al*., 2016). In Brazil, it is considered a neglected disease, with high prevalence and mortality (593,015 and 144,078 cases in 2015, respectively) (Lotufo *et al*., 2017). Indeed, the risk of dying prematurely due to stroke in Brazil is one of the highest in the world (Lotufo and Benseñor, 2009; Lotufo, 2015).

The involvement of CS in cerebrovascular disease has been reported by several studies, due to its close relationship with the coagulation cascade, which is suggested as a therapeutic target (Kramer *et al*., 2000; Mocco *et al*., 2006; Arumugam *et al*., 2009; Orsini *et al*., 2014; Larsen *et al*., 2018; Dobó *et al*., 2018; Ahmad *et al*., 2019; Ma *et al*., 2019). The LP probably plays a more important role than other complement pathways in cerebrovascular disease, possibly related to the role of MASP-1 in activating the coagulation cascade, leading to the formation of clots and/or recruiting leucocytes through pro-inflammatory factors, resulting in thrombosis and consequent tissue ischemia (Orsini *et al*., 2014; Dobó *et al*., 2018) (Figure 5).

Some independent studies (Cervera *et al*., 2010; Osthoff *et al*., 2011; Orsini *et al*., 2012; De La Rosa *et al*., 2014; Neglia *et al*., 2019, 2020) found a beneficial effect of MBL deficiency in stroke patients, improving prognosis and reducing C3, C4, and CRP levels, as well as the proinflammatory cytokine profile, neurological deficits and extent of brain lesions, although MBL may also be necessary at later stages, for the tissue recovery process (Ducruet *et al*., 2011). Besides that, ficolins also appear to play an important role in the severity of ischemia and in subarachnoid aneurysmal hemorrhage (SAH). Ficolin-2 and ficolin-3 occur at lower levels in ischemic stroke patients, due to their consumption in the acute phase of the pathology, and low ficolin-3 levels are associated with greater SAH severity (Füst *et al*., 2011; Orsini *et al*., 2014; Zanier *et al*., 2014). Orsini *et al*. (2016) demonstrated that MASP-2 contributes critically to tissue injury in an animal model of cerebral ischemia, probably due to an increase in C3 deposition in this area. Nevertheless, although MBL sufficient genotypes increased the odds for cerebral ischemia in humans, no protective association was found with the deficiency-causing *MASP2* p.D120G polymorphism, probably due to its low frequency (no other *MASP2* variant was investigated) (Cervera *et al*., 2010). Double *Masp1/Masp2* knock-out ischemic mice were also not protected (Orsini *et al*., 2016). In a study carried out in the Armenian population, it was found that patients with ischemic stroke had higher proteolytic activity of MASP-1 and MASP-2, than controls (5.8 and 3.7 times more, respectively) (Tsakanova *et al*., 2018). Taken together, the evidence warrants further investigation with MASP levels and polymorphisms (Tables 1 and 2) in the susceptibility to cerebral vascular ischemia, especially within the context of COVID-19 disease.

## Concluding remarks

The pathophysiology of COVID-19 presents systemic hyper inflammation and hypercoagulation, affecting several organs (Berlin *et al*., 2020). Molecules that play a role in both inflammation and coagulation can be considered effective therapeutic targets for COVID-19. MASPs (mainly MASP-1) are key elements in the interaction between the complement system and blood coagulation. Increased levels of MASPs are associated with comorbidities of COVID-19 (CVD, CKD, diabetes, COPD and cerebrovascular diseases), and are associated with exacerbated inflammation and activity of the coagulation cascade. Furthermore, MASP-2 is suggested to interact with the N protein of SARS-COV-2, connecting the LP directly with viral recognition. Thus, the understanding of COVID-19 pathogenesis may benefit from studies of MASPs and their associations with risk factors, and of the role played by these molecules at the crossroad between the complement and coagulation systems. We propose that MASPs can be explored as biomarkers for disease progression, since the lectin pathway may be the first response against COVID-19. Furthermore, studies investigating *MASP1* and *MASP2* polymorphisms that may modulate the levels of MASPs in COVID-19 patients, can contribute to a greater understanding of the disease. Based on the promising results of Narsoplimab treatment, we suggest that pharmacological therapies aiming to inhibit MASPs would be effective in the treatment of COVID-19 and associated comorbidities.
